# Effect of reverse Trendelenburg position and positive pressure ventilation on safe non-hypoxic apnea period in obese, a randomized-control trial

**DOI:** 10.1186/s12871-023-02128-7

**Published:** 2023-06-08

**Authors:** Etienne J. Couture, Antony Carrier-Boucher, Steeve Provencher, Issam Tanoubi, Simon Marceau, Jean S. Bussières

**Affiliations:** 1 Department of Anesthesiology and Critical Care, Laval University, Quebec, Canada; 2 Department of Anesthesiology, Institut Universitaire de Cardiologie et de Pneumologie de Québec – Université Laval, 2725, Chemin Sainte-Foy, Québec, QC G1V 4G5 Canada; 3 Department of Anesthesiology, Hôpital Sacré-Cœur, CIUSSS Nord de L’Île de Montréal, Montréal, Canada; 4 Department of Pneumology, Institut Universitaire de Cardiologie et de Pneumologie de Québec– Université Laval, Quebec, Canada; 5 Department of Anesthesiology, Centre Intégré Universitaire de Santé Et de Services Sociaux de L’Est-de-L’Île-de-Montréal, University of Montreal Medical Simulation Center (CAAHC), Montréal, Canada; 6 Department of Surgery, Institut Universitaire de Cardiologie , et de Pneumologie de Québec – Université Laval, Quebec, Canada

**Keywords:** Preoxygenation, Positioning, Obesity, Safety, Positive Pressure

## Abstract

**Purpose:**

There is an elevated incidence of hypoxemia during the airway management of the morbidly obese. We aimed to assess whether optimizing body position and ventilation during pre-oxygenation allow a longer safe non-hypoxic apnea period (SNHAP).

**Methods:**

Fifty morbidly obese patients were recruited and randomized for this study. Patients were positioned and preoxygenated for three minutes in the ramp position associated with spontaneous breathing without additional CPAP or PEEP (RP/ZEEP group) or in the reverse Trendelenburg position associated with pressure support ventilation mode with pressure support of 8 cmH_2_O and an additional 10 cmH_2_O of PEEP while breathing spontaneously (RT/PPV group) according to randomization.

**Results:**

The SNHAP was significantly longer in the RT/PPV group (258.2 (55.1) vs. 216.7 (42.3) seconds, *p* = 0.005). The RT/PPV group was also associated to a shorter time to obtain a fractional end-tidal oxygen concentration (FEtO_2_) of 0.90 (85.1(47.8) vs 145.3(40.8) seconds, *p* < 0.0001), a higher proportion of patients that reached the satisfactory FEtO_2_ of 0.90 (21/24, 88% vs. 13/24, 54%, *p* = 0.024), a higher FEtO_2_ during preoxygenation (0.91(0.05) vs. 0.89(0.01), *p* = 0.003) and a faster return to 97% oxygen saturation after ventilation resumption (69.8 (24.2) vs. 91.4 (39.2) seconds, *p* = 0.038).

**Conclusion:**

In the morbidly obese population, RT/PPV, compared to RP/ZEEP, lengthens the SNHAP, decreases the time to obtain optimal preoxygenation conditions, and allows a faster resuming of secure oxygen saturation. The former combination allows a more significant margin of time for endotracheal intubation and minimizes the risk of hypoxemia in this highly vulnerable population.

**Trial registration:**

NCT02590406, 29/10/2015.

## Introduction

Obesity prevalence is rising worldwide, leading to an increasing number of obese patients needing surgical interventions [1, 2]. During these interventions, this population has a higher risk of suffering from severe airway management complications, mainly hypoxemia, than non-obese patients due to a higher incidence of difficult mask ventilation and tracheal intubation [3–5]. In addition, the morbidly obese population has a reduced safe non-hypoxic apnea period (SNHAP), defined by the time interval after the onset of apnea before hypoxemia occurs, primarily due to higher oxygen consumption and a reduced functional residual capacity (FRC) [6, 7]. Thus, to minimize the risk of hypoxemia in these patients, it is crucial to optimize the preoxygenation period to allow more time for the anesthesiologist to secure the airway.

Various preoxygenation methods have been suggested to lengthen the SNHAP in the obese population. To increase the FRC and the oxygen reserve, several authors suggested optimizing the operating table position during preoxygenation [8–10], while others suggested preoxygenation with positive pressure ventilation [11, 12]. These strategies were more effective in prolonging the SNHAP than the supine position and the absence of positive pressure. We recently documented the effect on FRC of various combinations of position; supine, beach-chair and reverse Trendelenburg as well as ventilation strategies; with or without positive pressure support. Among the six tested combinations, the association of reverse Trendelenburg position with non-invasive positive pressure ventilation was the one associated with the most significant increase in FRC in morbidly obese patients [13]. However, the effects of this combined strategy on oxygenation parameters have never been assessed experimentally.

This study was thus designed to compare the effects of reverse Trendelenburg position associated with non-invasive positive pressure ventilation compared to the traditional preoxygenation in ramp position associated with spontaneous breathing on SNHAP in morbidly obese patients. We hypothesized that the former would improve oxygenation parameters in morbidly obese patients undergoing general anesthesia.

## Methods

The study protocol of this randomized control trial had been approved by the research ethic board of our institution (Institut Universitaire de Cardiologie et de Pneumologie de Québec, Canada, (CER21211) and registered (Clinicaltrial.gov NCT02590406) in October of 2015, before the enrolment of the first patient. This research protocol had been performed in accordance with the Declaration of Helsinki and all methods were carried out in accordance with relevant guidelines and regulations. Patients were recruited at the anesthesia preoperative clinic. All patients provided informed written consent.

### Study objective and outcomes

The main objective of this trial was to evaluate, in a clinical setting, the effect of a 3-min preoxygenation in the recommended combination of ramp position associated with spontaneous breathing without additional continuous positive airway pressure (CPAP) or positive end-expiratory positive pressure (PEEP) (RP/ZEEP (spontaneous ventilation without positive pressure ventilation) group) to the reverse Trendelenburg position used in association with non-invasive positive pressure ventilation with pressure support of 8 cm H_2_O on an additional 10 cm of H_2_O of PEEP via the ventilator circuit while breathing spontaneously (RT/PPV group). The primary outcome measure was the SNHAP, defined as the interval between the induction of anesthesia and the occurrence of oxygen saturation (SpO_2_) of 92%. Secondary outcome measures included: 1) the time to obtain a FetO_2_ of 0.90; 2) the proportion of patients that reached 0.90 of FetO_2_; 3) the maximal FetO_2_ during preoxygenation; 4) the time needed to return to a SpO_2_ of 97% after ventilation resumption and; 5) the mean arterial pressure during the experimental period.

### Population

Patients 21 years or older, scheduled for bariatric surgery with a minimal body mass index (BMI) of 40 kg·m^−2^, having a waist circumference of ≥ 115 cm for women and ≥ 130 cm for men, were eligible. We excluded patients with facial hair, asthma, moderate and severe chronic obstructive pulmonary disease (defined as a forced expiratory volume in one second (FEV_1_) less than 80% of expected value and an FEV_1_/forced vital capacity less than 0.7), severe cardiac failure (defined as a New York Heart Association classification of IV), high risk of gastroesophageal regurgitation (defined as symptomatic gastroesophageal reflux disease despite medication or previous gastroesophageal surgery), pregnancy, active tobacco use, already known or suspected difficult airway by the attending anesthesiologist and significant craniofacial abnormalities [14].

### Study design

Patients fasted for 8 h and received oral ranitidine 150 mg the evening before and the morning of their bariatric surgery. After the intravenous placement and application of standard American Society of Anesthesiologist (ASA) physiological monitors, the attending anesthesiologist evaluated the airway to decide if the patient could participate in the study. The patient was randomized if the anesthesiologist judged the patient’s airway not at risk of difficult mask ventilation or intubation. Sequentially numbered opaque envelopes prepared by an independent research assistant, in clusters of ten in a 1:1 ratio, were used for randomization between the two groups (RP/ZEEP versus RT/PPV).

**Fig. 1:**
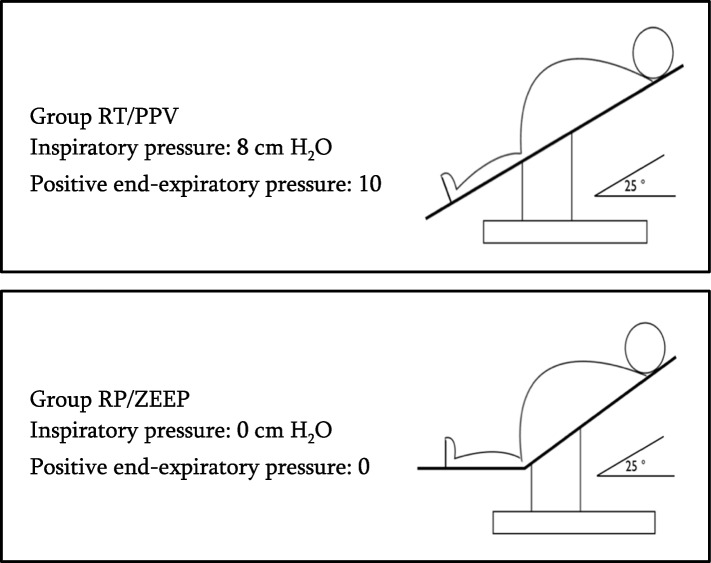
Design of the intervention. Both groups had a 3-min preoxygenation with an inspiratory fraction of oxygen (FiO_2_) set to 1.0 and the fresh gas flow set to 18 L/min from an anesthesia machine using a soft snorkel-type mouthpiece with a nose clip instead of a facemask to prevent air leaks. Abbreviation: PPV: positive pressure ventilation, RP: ramp position, RT: reverse Trendelenburg position, ZEEP: zero end-expiratory pressure ventilation

Before the start of the preoxygenation, for patients in group 1 (RP/ZEEP), the operating table (Universal operating table, Alphamaxx, Maquet, Rastatt, Germany) was set in a 25º beach-chair position (upper body portion was tilted upward at a 25º angle). The anesthesia ventilator (Dräger Primus, Lübeck, Germany) was set in spontaneous ventilation without any positive pressure ventilation with the adjustable pressure-limiting valve sets in the open position. The beach chair position used in our study is similar to the ramp position [15], being probably the most frequently used for the induction of bariatric patients. Patients in group 2 (RT/PPV) were positioned in a 25º reverse Trendelenburg position (the entire operating table tilted 25º). The anesthesia ventilator was set to a pressure support ventilation mode with an inspiratory pressure of 8 cm H_2_O and a positive end-expiratory pressure (PEEP) of 10 cm H_2_O [13]. Table angles were measured at the hip hinge with a digital angle level (ROK, Burnaby, British Colombia, Canada) (Fig. 1).

In both groups, the inspiratory fraction of oxygen was set to 1.0, and the fresh gas flow was set to 18 L/min. The ventilatory interface used during preoxygenation was a soft mouthpiece (Airlife adult flexible mouthpiece, CareFusion, San Diego, CA, USA) with a nose clip instead of a facemask to prevent air leaks, given the high tolerability previously observed with this device in a similar study population [13]. During the preoperative period, we gave explanation to the patient why and how to use this mouthpiece. The ventilator circuit was then connected to the mouthpiece and patients from both groups were asked to breathe normally during the 3-min preoxygenation period.

### Measurements

Before the entry into the operating room, we collected the following preoperative data: age, sex, height, weight, neck, hip and waist circumference, Mallampati and ASA score, FEV_1_, presence of obstructive sleep apnea and the use of home continuous positive airway pressure (CPAP) treatment for sleep apnea.

From the moment the ventilator circuit was connected to the mouthpiece, inspiratory and expiratory fractions of oxygen, carbon dioxide and minute ventilation were continuously monitored and displayed on the anesthesia machine (Dräger Primus, Lübeck, Germany). The time required to obtain a FetO_2_ of 0.90 was first recorded, with the final FetO_2_ value and the mean minute ventilation after the 3-min preoxygenation period. At the end of preoxygenation, anesthesia was induced with sufentanil 0.2–0.3 µg kg^−1^, propofol 1–3 mg kg^−1^ and succinylcholine 1–1.5 mg kg^−1^ or rocuronium 1 mg kg ^−1^, followed by a continuous infusion of propofol 100 µg kg^−1^ min^−1^. Lean body weight was used for the calculation of propofol doses [16]. After the induction of anesthesia, the mouthpiece was removed. No ventilation was provided to the patient. The attending anesthesiologist performed the orotracheal intubation using either direct laryngoscopy (Macintosh or Miller blade) or videolaryngoscopy (C-MAC, Storz, Tuttlingen, Germany) when the patient was paralyzed according to the neuromuscular monitor (Life-Tech, EZ Stim II, Stafford, TX, USA). After the intubation, the attending anesthesiologist confirmed the adequate tube position with a fiberscope and kept the circle-breathing system disconnected. The duration of the SNHAP (until SpO_2_ had fallen to 92%) was recorded. Then, the breathing system was reconnected. The anesthesia ventilator was set to volume-controlled ventilation at a frequency of 20 ventilations per minute, a tidal volume of 8 mL kg^−1^ of predicted ideal body weight and PEEP of 10 cm H_2_O until SpO_2_ reached 97%. We recorded the blood pressure every minute and the minimal SpO_2_ reached after resuming the ventilation.

#### Statistical analysis

The primary endpoint was analyzed using one-way ANOVA and expressed using mean (SD), after ensuring the normal distribution with a D’Agostino-Pearson omnibus normality test. We used Kaplan–Meier curves to illustrate the length of stay with greater SpO2 than 92% for both groups. We performed a log-rank (Mantel-Cox) test to compare them. Secondary endpoints were analyzed using one-way ANOVA to compare groups. The univariate normality assumption was verified with the Shapiro–Wilk tests on the error distribution from the statistical model. Brown and Forsythe's variation of Levene's test statistic was used to verify the homogeneity of variances. Time for SpO_2_ > 97% (seconds) was log-transformed to fulfill to normality and variance assumptions. Characteristic variables expressed in percentage were analyzed using Chi-Square or Fisher’s exact test. The results were considered significant with *P* values < 0.05. All analyses were conducted using the statistical package SAS, version 9.4 (SAS Institute Inc, Cary, NC, U.S.A.) and R (R Core Team (2016), Foundation for Statistical Computing, Vienna, Austria).

We calculated our sample size using data from a previous study [13], where the authors found a difference in the FRC of 465 (473) mL (21%) between reverse Trendelenburg with non-invasive positive pressure ventilation and beach-chair position without positive pressure ventilation. Assuming that FRC is representative of the oxygen reserve available during SNHAP, a difference of 21% in the apnea time would be expected. Considering a type I error of 5% and power of 80%, 17 patients per group were needed. To overcome post-randomization exclusions, 25 patients were randomly assigned to each group.

## Results

**Fig. 2:**
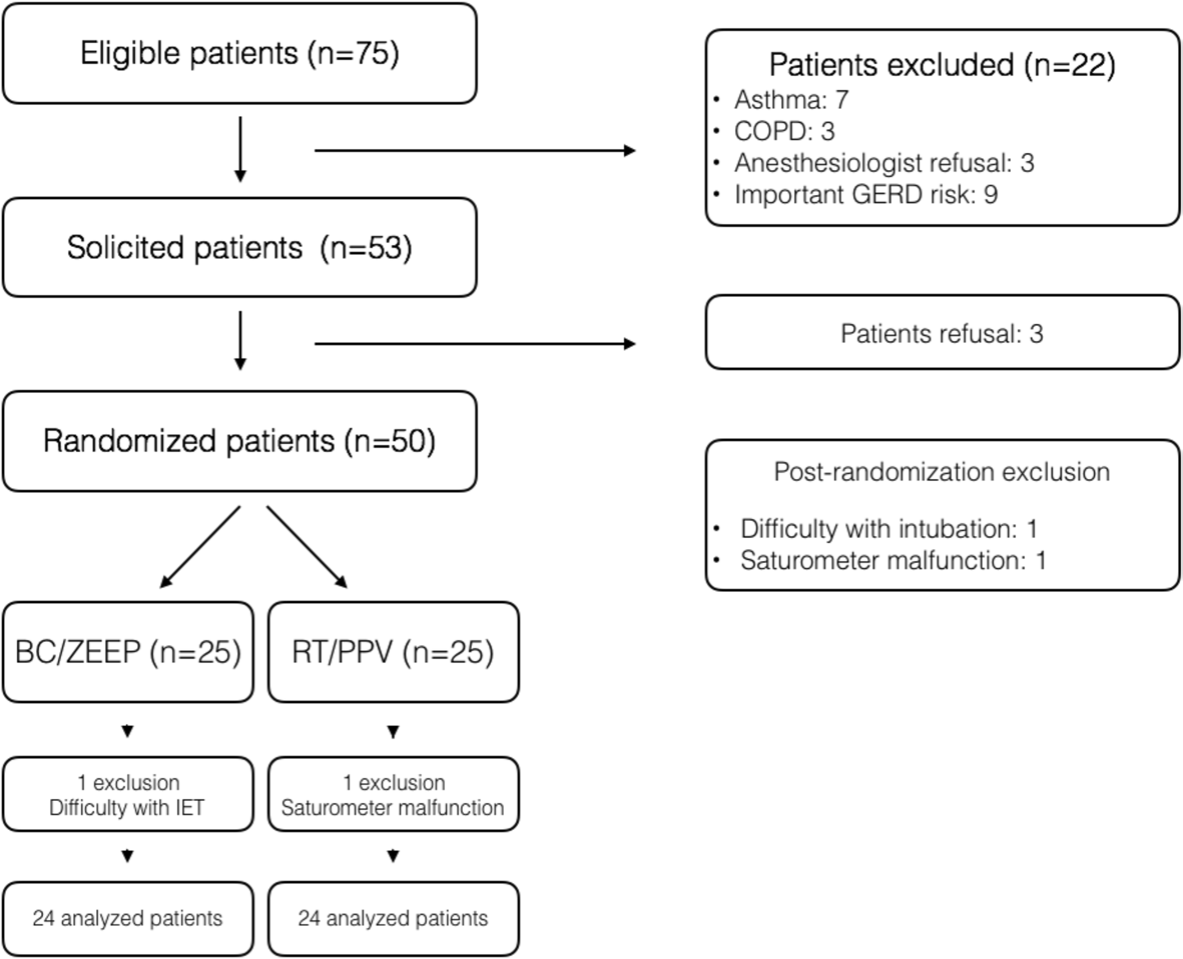
Consort flowchart. *Abbreviations: RP/ZEEP*: Beach-chair position without positive pressure, *COPD*: chronic obstructive pulmonary disease, *GERD*: gastroesophageal reflux disease, *IET*: insertion of endotracheal tube, *RT/PPV*: Reverse Trendelenburg position associated with positive pressure ventilation

Fifty (50) patients were recruited from September until December 2015. One patient in the RP/ZEEP group was excluded because of an unexpected airway management difficulty leading to mask ventilation before definitive intubation. One patient in the RT/PPV group was excluded because of a pulse oximeter malfunction. Thus, 48 patients were included in the analysis (Fig. 2).

The patients’ characteristics were similar (Table 1).

**Table 1:** Demographic data

	**RP/ZEEP (*n* = 24)**	**RT/PPV (*n* = 24)**
Age (years)	40 (9)	46 (11)
Gender (M:F)	08:16	06:18
Weight (kg)	131.7 (21.2)	129.0 (20.8)
Height (m)	1.7 (0.1)	1.6 (0.1)
BMI (kg·m^−2^)	47.9 (6.3)	47.3 (5.2)
Waist circumference (cm)	138.4 (15.0)	133.5 (14.7)
Hip circumference (cm)	142.8 (14.8)	136.4 (11.1)
Waist: Hip ratio	1.0 (0.1)	1.0 (0.1)
Neck Circumference (cm)	43.9 (5.9)	44.9 (4.8)
FEV_1_ (liters)	2.9 (0.8)	2.8 (0.6)
History of sleep apnea (n)	12	11
Epworth score	5.4 (3.9)	6.9 (3.7)
Stop-Bang score	3.3 (1.2)	3.7 (1.1)
Use of CPAP at home (n)	9	10
Mallampati classification (1:2:3:4)	4:11:8:1	3:14:7:0
ASA status (1:2:3:4:5)	0:0:24:0:0	0:0:24:0:0
Surgery (Gastrectomy: biliopancreatic diversion)	17:7	13:11

Results are presented as mean (SD), unless otherwise specified

*Abbreviations:* *ASA* American Society of Anesthesiologists, *RP/ZEEP* Ramp Position/Zero End-Expiratory Pressure, *BMI* Body Mass Index, *CPAP* Continuous Positive Airway Pressure, *FEV*_*1*_ Forced expiratory volume in 1 s, *RT/PPV* Reverse Trendelenburg/Positive Pressure Ventilation

All the patients tolerated very well the mouthpiece oxygenation. All patients were intubated before the SpO_2_ of 92% was reached, and all endotracheal tubes were adequately positioned upon fiberscopic assessment.

**Fig. 3:**
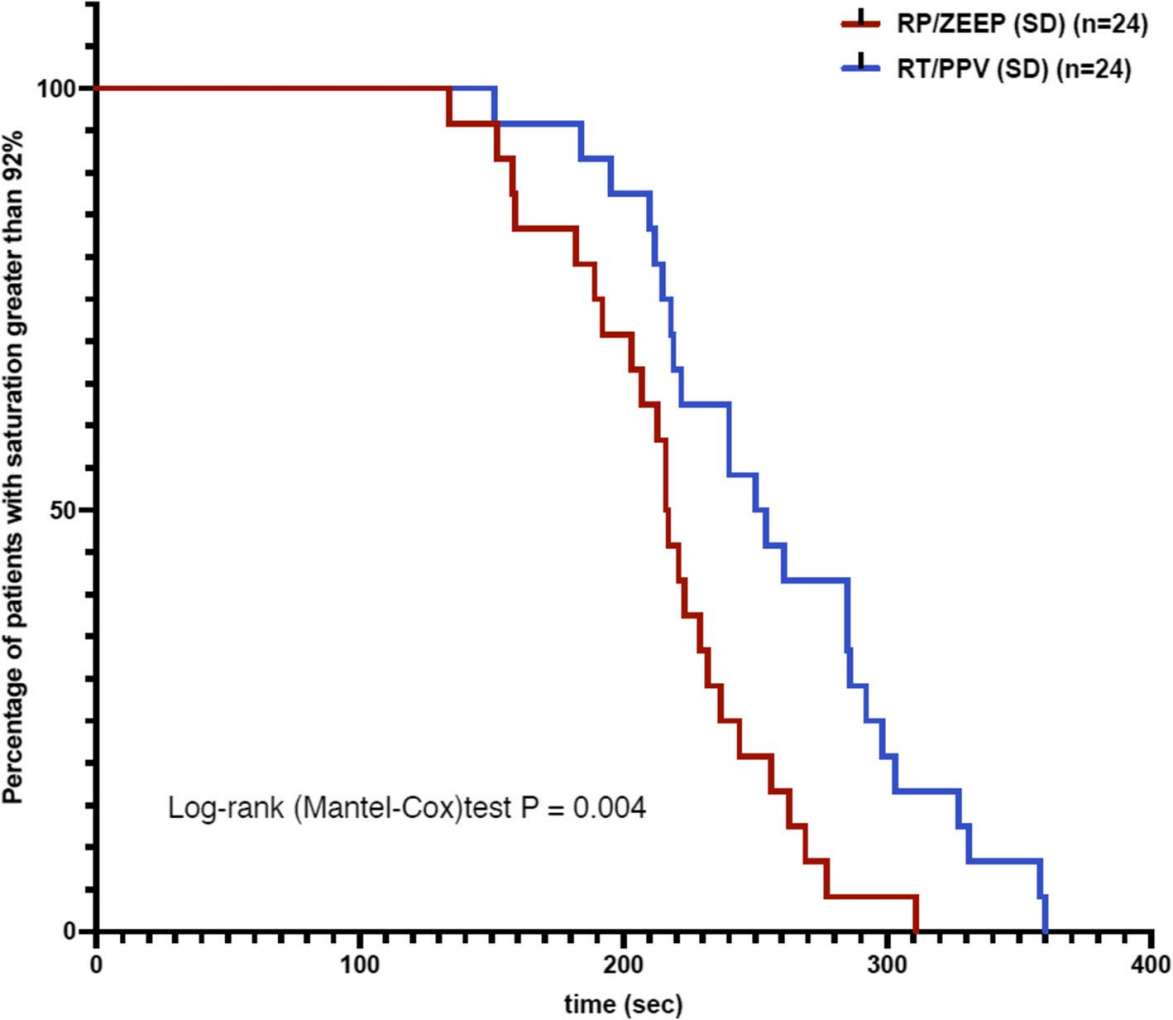
Kaplan–Meier curves. The Kaplan–Meier curves illustrate the length of stay with greater SpO2 than 92% for both groups. Abbreviation: PPV: positive pressure ventilation, RP: ramp position, RT: reverse Trendelenburg position, ZEEP: zero end-expiratory pressure ventilation

The SNHAP was longer in the RT/PPV group than in the RP/ZEEP group (258 (55.1) vs. 216.7 (42.3) seconds, *p* = 0.0053) (Table 2 and Fig. 3).

**Table 2:** Results

	**RT/PPV** **(*n* = 24)**	**RP/ZEEP** **(*n* = 24)**	***P***
**Primary endpoint**			
Safe non-hypoxic apnea period (seconds)	258.2 (55.1)	216.7 (42.3)	0.0053
**Secondary endpoint**			
Time for FetO_2_ > 0.9 (seconds)	85.1 (47.8)	145.3 (40.8)	< 0.0001
Maximal FetO_2_ (%)	91 (5)	89 (1)	0.0003
Time for SpO_2_ > 97% (seconds)	69.8 (24.2)	91.4 (39.2)	0.0384
Proportion of patients that reached FeO_2_ of 0.90 (%)	21/24 (88)	13/24 (54)	0.0243

Results are presented as mean (SD), unless otherwise specified

*Abbreviations:* *RP/ZEEP* Ramp Position/Zero End-Expiratory Pressure, *FetO*_*2*_ fractional end-tidal oxygen concentration, *RT/PPV* Reverse Trendelenburg/Positive Pressure Ventilation, *SpO*_*2*_ pulsed oxygen saturation

During the preoxygenation phase, patients reached a FetO_2_ of 0.90 faster in the RT/PPV group than in the RP/ZEEP group and reached a higher final FetO_2_ after three minutes. Moreover, a higher proportion of patients in the RT/PPV group reached the satisfactory 0.90 of FetO_2_. Minute ventilation was higher in the RT/PPV group (10.9(3.2) vs. 7.6(2.4) L/min, *p* = 0.0004). After the resumption of ventilation, there was no difference in the minimal SpO_2_ (85.3(1.8) vs. 83.6(7.9) %, *p* = 0.9102) but it took less time for the RT/PPV group to return to a 97% SpO_2_ in comparison to the RP/ZEEP group.

**Fig. 4:**
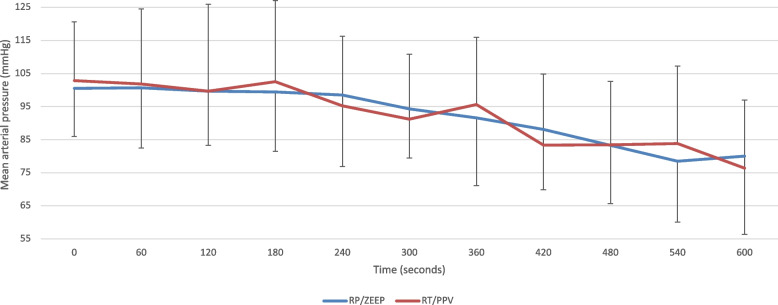
Mean Arterial Pressure. Mean arterial (mmHg) pressure, for both groups, during the first 10 min following the beginning of the pre-oxygenation including the periods of anesthesia induction and the tracheal intubation. *P* = 0.8893

The mean arterial pressure was similar in both groups during the first 10 min of the experimental period and no vasoactive drugs were needed (Fig. 4).

Post operatively, asking to the patient, no one complained about discomfort with the use of the mouthpiece and nose-clamp.

## Discussion

This randomized control trial reveals that the SNHAP is longer in the morbidly obese population when the preoxygenation and induction of anesthesia are done using the RT/PPV combination compared to a traditional combination of RP/ZEEP. These results were substantiated by secondary outcome measures confirming that the RT/PPV combination was also associated with improvements in all oxygenation parameters assessed. Our results support the use the RT/PPV combination for the preoxygenation of morbidly obese patients undergoing generalized anesthesia.

Preoxygenation in two different head-up positions (reverse Trendelenburg and beach-chair) has already been compared in the context of SNHAP optimization for the morbidly obese. In their preliminary study, Boyce et al*.* found that the reverse Trendelenburg position was superior to the supine and beach-chair positions [10]. Interestingly, the SNHAP measured after preoxygenation in the beach-chair position was shorter in the study by Boyce et al*.* compared to the present study (153(63) vs 216(42) seconds). This can be explained by differences in preoxygenation protocols, as well as patients’ characteristics (e.g., mean BMI of 53(9) vs. 48(6)), knowing that there is a reverse linear relation between BMI and SNHAP [6, 17].

Conversely, using positive pressure ventilation during preoxygenation gave conflicting results on SNHAP in previous studies. Using PEEP [11] or PPV, [18] some groups did not observe any favorable changes in SNHAP [12] or a higher PaO_2_ at the end of the preoxygenation [19]. Since the position and ventilation strategies were not individually compared in the present study, it remains unknown whether the beneficial effects of RT/PPV compared to RP/ZEEP are related to differences in position, ventilation strategy or their combination. However, the use of PPV reduces atelectasis [20] and increases FRC, [13] thus likely decreasing intrapulmonary shunt [21] and leading to better use of the expanded oxygen store. Moreover, the use of PPV has been shown to have additive effects on FRC amongst patients in RT.^13^ Interestingly, several authors had previously studied the impact of PPV during preoxygenation on gastric distention and concluded that it is safe to use PPV unless the peak inspiratory pressure goes above 20 cmH_2_O [11]. The main advantage of the reverse Trendelenburg position over the ramp position is to reduce the transmission of intra-abdominal pressure on the diaphragm responsible for its altered excursion and ventilation impairment [22]. Also, it lowers intragastric pressure [23] and the risk of regurgitation in this high-risk population [24].

Interestingly, the increase in SNHAP found in this study is consistent with the hypothesis that the SNHAP directly correlates with patients’ FRC. Assuming that the FRC is composed of 90% oxygen at the end of the preoxygenation, the 465 mL difference in FRC previously observed between the RP/ZEEP and the RT/PPV groups would result in a difference of 418 mL in oxygen content between groups. Estimating a total body weight of 130 kg, a basal oxygen consumption (VO_2_) of 3.5 mL kg^−1^ min^−1^ (i.e., 455 mL min^−1^), the preoxygenation using an RT/PPV combination would be expected to result in a 55-s increase in SNHAP which is very close to the 42-s difference observed in the present study.

Patients in the RT/PPV group also improved other oxygenation parameters. Indeed, FetO_2_ > 0.9 was reached in a higher proportion and faster than in patients in the RP/ZEEP group. This result can be explained by higher minute ventilation that allowed a faster nitrogen washout. This higher minute ventilation is owed to the PPV that helped patients of this group to increase their minute ventilation with minimal effort. Because patients did not receive any sedation before the anesthesia induction, there should be no difference in the airway patency or the respiratory drive between both groups. We think the reverse Trendelenburg position might also have helped by increasing the total thoracic compliance. After intubation, when ventilation was resumed following reaching SpO_2_ of 92%, the shorter time to return to a SpO_2_ of 97% observed in the RT/PPV group might also be explained by better ventilatory mechanics owing to the RT position. The reduction in atelectasis might also explain better V/Q matching after using PEEP, which is known to remain many minutes after the PEEP has been withdrawn [25]. In this way, if difficulties with airway management were to occur and the patient’s saturation was to decrease, it would be faster to return to a normal SpO_2_ value with the former combination.

The mouthpiece and nose-clamp used in the present study is a clinical routine for pulmonary function testing. We used this technic in one of our previous studies in awake bariatric subjects and during a pilot study in the same context that the reported study (13). We observed a high tolerability with this device in a similar study population. We could speculate that the impact of this use was probably low in the results of this study.

This study has several limitations. The increase of the oxygenation was achieved while changing operating table position [8–10] or using PPV, [11, 12] both shown superior to the supine position or the absence of positive pressure. Therefore, the specific impacts of the combination of positional and ventilatory strategies on SNHAP remain elusive. Notably, the decision to perform a combination of interventions was based on the previous observations that RT/PPV was associated with the optimal FRC increase compared to either RT alone, PPV alone, or RP/ZEEP. Accordingly, clinicians should strongly consider the use of the combined RT/PPV to benefit from the prolonged SNHAP demonstrated in this study. Secondly, the attending anesthesiologists were not blind to the group assignment. However, since the outcomes were objective measurements, the impact on results was minimized. Another limitation of the study is the lack of standardization in the neuromuscular blocking agent. The use of succinylcholine enhances the oxygen consumption in animal models due to related muscle fasciculation, [26] but not in humans [27]. If the effect on oxygen consumption were to be significant in humans, it would have affected the difference in SNHAP between the groups due to difference in succinylcholine use.

## Conclusion

In the morbidly obese population, RT/PPV lengthens the SNHAP, decreases the time to obtain optimal preoxygenation conditions, and allows a faster resuming of secure oxygen saturation compared to RP/ZEEP, the former combination thus allows a more significant margin of time for endotracheal intubation and minimizes the risk of hypoxemia in this highly vulnerable population.

## Data Availability

The datasets used and/or analysed during the current study available from the corresponding author on reasonable request.

## Abbreviations

ASA: American Society of Anesthesiologists
RP/ZEEP: Ramp position without positive pressure ventilation
BMI: Body mass index
COPD: Chronic obstructive pulmonary disease
CPAP: Continuous positive airway pressure
EtO_2_: End-tidal oxygen concentration
FetO_2_: Fractional end-tidal oxygen concentration
FEV_1_: Forced expiratory volume in one second
FRC: Functional residual capacity
GERD: Gastroesophageal reflux disease
IET: Insertion of endotracheal tube
PEEP: Positive end-expiratory pressure
PPV: Positive pressure ventilation
RT: Reverse Trendelenburg
RT/PPV: Reverse Trendelenburg position associated with positive pressure ventilation
SNHAP: Safe non-hypoxic apnea period
SpO_2_: Oxygen saturation
VO_2_: Oxygen consumption
ZEEP: Spontaneous ventilation without positive pressure ventilation
